# Can Physical Activity Intensity Condition Anxiety, Mental Hyperactivity, and Resilience in Higher Education Students?

**DOI:** 10.3390/healthcare13202566

**Published:** 2025-10-11

**Authors:** Rubén Fernández-García, Cristina González-Forte, María Rosa Ortega-Lasheras, Eduardo Melguizo-Ibáñez

**Affiliations:** 1 Department of Nursing, Physiotherapy and Medicine, University of Almeria, 04120 Almeria, Spain; 2 Torrecárdenas University Hospital, 04009 Almeria, Spain; 3 Department of Specific Didactics, University of La Laguna, 38204 Tenerife, Spain; emelguiz@ull.edu.es

**Keywords:** physical activity, mental hyperactivity, anxiety, resilience, structural equation model

## Abstract

**Background/Objectives**: Scientific literature has demonstrated the positive effects of physical activity in college students. The research seeks to examine the relationships of light, moderate and vigorous physical activity on resilience, mental hyperactivity and anxiety. **Methods**: An explanatory and cross-sectional study was carried out. A sample of 2305 university students belonging to different university degrees participated in the study. The following questionnaires were used: International physical activity and mental hyperactivity. The Connor and Davidson Resilience Scale (CD-RISC) and the Depression, Anxiety and Stress Scale (DASS-21) were also used. **Results**: Regarding the relationship between light physical activity and anxiety, a weak but significant relationship was observed (*β* = 0.027, *p* < 0.05). A weak but significant relationship was also observed between light physical activity and mental hyperactivity (*β* = 0.044, *p* < 0.05). **Conclusions**: The promotion of moderate-vigorous physical activity together with the development of personal resilience can be effective tools to reduce anxiety and mental hyperactivity in the university population.

## 1. Introduction

Nowadays, one of the main problems affecting today’s university society is anxiety [1]. Physical and psychological illnesses may be due to a progressive load of stressors, which alter over time, the Central Nervous System [2]. Chronic anxiety can eventually facilitate such serious health problems as cancer, due to its potentiating effect on the release of stress hormones [2]. These hormones interact with inflammatory cell receptors and the activation of signalling pathways that enhance the activation of Kappa B cells (NF-κB) [2,3]. One of the different tools to combat anxiety and stress is the regular practice of physical activity [4].

Different studies have highlighted that physical activity practice is an effective tool to combat negative emotional states [5]. Likewise, one of the factors to be regarded in the physical activity practice is the intensity [6]. Intensity is defined as the rate or level of effort at which the physical task is performed [6]. Different investigations have concluded that the intensity at which physical activities are performed conditions the effects on physical and mental health [6]. Activities performed at a vigorous intensity cause the release of different neurotransmitters [7]. The secretion of these neurotransmitters causes a decrease in the anxiety levels of students [8]. Also, physical activity practice causes a decrease in the Default Neural Network [6]. This leads to a decrease in the levels of mental hyperactivity [6].

Mental hyperactivity is directly related to anxiety levels and the so-called Default Neural Network [9]. This brain networks supports advanced cognitive functions, including episodic projection, theory of mind and autobiographical processing [10]. The Default Neural Network is a highly structured system, fundamental in decision making and brain organization [9,11]. If this system is altered by excessive stressors, a state of mental hyperactivity can be generated [6,9,11]. This phenomenon is highly complex and, in addition, structures such as the amygdala and hypothalamus, among others, are also involved [11]. Therefore, it is a network directly related to problem solving, especially in situations of high stress and anxiety [11]. It should be noted that the Default Neural Network is deactivated especially in situations that require focused attention [6,9,11]. Therefore, physical activity and exercise practice avoid situations of mental hyperactivity [6].

Therefore, a high level of mental hyperactivity causes students to face high levels of anxiety and stress [12,13]. It is very important to prepare university students to manage difficult moments and frustration [11]. In this sense, resilience, or the ability to recover and become stronger after adversity, is particularly relevant [14]. People with low resilience, where sometimes college students can be included, are more vulnerable to social events such as rejection, and have more negative emotions, favouring the tendency to mental hyperactivity [14]. On the contrary, people with high resilience present a greater number of psychological resources before problems and have less anxiety [15]. Physical activity is a useful tool for the development of resilience [16]. The practice of moderate-vigorous physical activity leads to better coping with stress [16], increases frustration tolerance [16] and regulates the nervous system [16]. All this leads to greater resilient behavior towards academic anxiety [16].

Students with low levels of resilience are more vulnerable to adverse social events, experience negative emotions more intensely, and tend to exhibit higher levels of mental hyperactivity [12,13,14]. Conversely, students with high levels of resilience have been found to have more psychological resources to cope with problems [15]. They also exhibit lower levels of anxiety [15]. It has been observed that regular physical activity performed at a moderate-vigorous intensity promotes the development of resilient behaviours [16], promoting the release of neurotransmitters that favour the deactivation of the Default Neural Network [6,7,8]. Thus, the combination of physical activity of adequate intensity allows students to manage frustration, regulate the nervous system, and maintain emotional and cognitive balance in the face of academic anxiety [16].

The original value of this research lies in the development and validation of a theoretical model that integrates physical activity intensity, resilience, anxiety and mental hyperactivity in university students, highlighting the differential effect of light, moderate and vigorous physical activity. Unlike prior studies that generally examined physical activity [14,15,16], this study demonstrates that light physical activity may increase anxiety and mental hyperactivity, whereas moderate and vigorous intensities are more beneficial in reducing them. Furthermore, it emphasises resilience as a protective factor that directly counteracts mental hyperactivity, offering a novel perspective on its role in emotional regulation and cognitive functioning.

Once the variables have been contextualized, the hypotheses of this research are the following:

**H.1.**  *Physical activity performed at a light, moderate or vigorous intensity will exert a negative relationship on anxiety and mental hyperactivity.*

**H.2.**  *Anxiety will be positively related to mental hyperactivity.*

**H.3.**  *Resilience will be negatively associated with mental hyperactivity.*

Finally, the objectives of the study are:

(a) To adjust and develop a theoretical model of physical activity intensity on resilience, mental hyperactivity and anxiety.
(b) To analyse the relationships of light, moderate and vigorous physical activity on resilience, mental hyperactivity and anxiety.

## 2. Materials and Methods

### 2.1. Design and Participants

The study was conducted in accordance with the STROBE statement [17]. The study design was exploratory and cross-sectional. Non-probability and convenience sampling was used. The initial sample consisted of 2350 participants, of whom 45 were eliminated for not responding adequately to the items. The final sample consisted of 2305 students (mean age = 25.49; standard deviation = 2.35), belonging to various universities in Andalucía. According to gender distribution, 1193 are male (51.75%) and 1112 are female (48.25%). Continuing with the distribution of students by field of study, this was distributed as follows: Social Sciences (n = 507; 21.99%), Sciences (n = 415; 18%), Engineering and Technology (n = 576; 24.99%), Arts and Humanities (n = 346; 15.01%) and Health Sciences (n = 461; 20.01%). In terms of employment status, most students are solely focused on their studies (n = 1498; 64.97%), while the rest combine academic work with employment (n = 807; 35.03%). The inclusion criteria established are as follows: (1) Be of legal age, (2) be studying for a university degree, (3) have no cognitive problems and (4) attend classes in person

### 2.2. Instruments and Variables

**International Physical Activity Questionnaire**: The original version of this instrument was developed by Carign et al. [18]. The version adapted into Spanish was used for this study [19]. It provides data related to the duration and frequency of physical activities at different intensity levels [19]. It includes seven questions designed to assess physical activity: intensity (light, moderate or vigorous), frequency (number of days per week) and duration (time per day). It demonstrated good internal consistency with Cronbach’s alpha of α = 0.76 and a MacDonald’s omega of was ω = 0.78.

**Depression, Anxiety and Stress Scale (DASS-21):** The original version of this instrument was developed by Lovibond and Lovibond [20]. The version adapted into Spanish was used for this study [21]. It consists of a total of 21 items that are evaluated using a Likert scale [21]. It has reported excellent psychometric properties in the general population, in adolescents and in university population. In the reliability analysis, the questionnaire has obtained an overall value of α = 0.917 for anxiety [21]. On this occasion, only the items corresponding to the anxiety scale have been used [21].

**Mental hyperactivity questionnaire:** It assesses the activation of the default neural network in the three months prior to completion. It consists of 10 items that are evaluated using a 5-point Likert scale. The higher the score, the greater the level of mental hyperactivity observed [9]. The final score is the sum of the scores for the 10 items, which provides an average value related to the individual’s overall mental hyperactivity. This questionnaire has been validated in university students [9] and only in Spanish. Adequate reliability values were obtained for this instrument (α = 0.89; ω = 0.91).

**Brief Resilience Scale CD-RISC:** The original version of this instrument was developed by Connor and Davidson [22]. For this study, the short version adapted into Spanish [23] was used. It consists of a 5-point Likert-type. Regarding psychometric properties, the test has unidimensional internal validity (CFI = 0.97, RMSEA = 0.05, SRMR = 0.03) and adequate internal consistency reliability (α = 0.85) [23].

### 2.3. Procedure

A Google Forms questionnaire was developed using the instruments previously described. After providing informed consent, university students who met inclusion criteria completed the questionnaire via link sent to their electronic devices. To verify the accuracy of responses, three items were intentionally repeated. If a participant gave inconsistent answer to these items, their data were excluded from the study. The responses of 45 participants were discarded. Data collection occurred from January 2024 to January 2025. Participation was entirely voluntary and preceded by informed consent. The study complied with the ethical principles outlined in the Declaration of Helsinki and was approved by the Ethics Committee of the University of Almería (EFM 419.25).

### 2.4. Data and Analysis

The statistical software IBM AMOS 23.0 was utilized to construct the structural equation model. Model fit was assessed using Increment Fit Index (IFI), Comparative Fit Index (CFI) and Normalized Fit Index (NFI). Acceptable values for these indices indicate values above 0.90 [21]. Additionally, the Root Mean Square Error of Approximation (RMSEA) was examined, with values below 0.08 considered indicative of a good fit [24].

**Figure 1:**
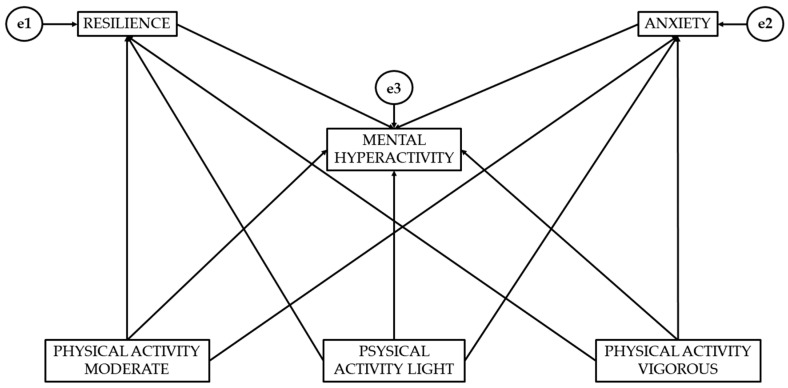
Theoretical representation of the structural equation model.

Figure 1 represents the model obtained with structural equations. A confirmatory structural equation analysis was performed to evaluate the adequacy of the proposed theoretical model. Physical activity intensity (light, moderate, and vigorous) was considered to act as exogenous variables. On the other hand, resilience, anxiety, and mental hyperactivity acted as endogenous variables since their variation can be explained by the intensity at which physical activity is performed.

The structural model includes error terms (e1, e2, and e3), which represent the unexplained variance of the endogenous variables. This allows us to assume that every endogenous variable is influenced not only by the variables that predict it, but also by unobserved factors, measurement errors or random variability [25]. In this way, the error terms allow us to estimate the relationship between the constructs more realistically, ensuring that the observed associations are interpreted as relationships subject to a portion of residual variance [26].

From a theoretical perspective, research has shown that the intensity at which physical activity is performed may be related to lower levels of anxiety [4] and higher levels of resilience [4,5,15]. Similarly, mental hyperactivity has been identified as a mediating mechanism in these associations. Likewise, the fit indices obtained indicate that the specified model adequately represents the data and constitutes the best approximation to the proposed relationships [24,25,26].

To perform the statistical analysis of the results, the IBM SPSS V29.02 statistical package was used. First, we proceeded to analyse the normality of the results. This analysis was carried out by means of the skewness and kurtosis values of each item [24,25]. The skewness value should range between −1.5 and 1.5. The kurtosis values should be between −3 and 3 [24,25]. Likewise, the reliability of the instruments used was evaluated through the Cronbach’s Alpha and McDonald’s Omega tests, establishing the reliability index at 95%.

## 3. Results

The fit indices obtained for the theoretical model developed were: X^2^ = 267.124; df = 4; IFI = 0.999, CFI = 0.900; NFI = 0.921; RMSEA = 0.072). The values obtained show a good fit of the theoretical model [21,22]. A descriptive and correlational analysis was performed (Table 1). All variables showed a normal distribution, as the asymmetry values ranged between −1.5 and 1.5 [24,25,27]. Similarly, the kurtosis values ranged between −3 and 3 [24,25,27]. The mean value for the anxiety variable was 2.1 ± 0.5. For mental hyperactivity, the mean was 2.2 ± 0.6. For resilience, the mean was 3.1 ± 0.5. The mean value for light physical activity was 338,296 ± 115.8, while for moderate and high-intensity physical activity it was 1756.823 ± 416.1 and 7856,308 ± 3895.9, respectively.

Based on the associations observed between variables, there is a significant negative correlation between anxiety and resilience (r = −0.35; *p* < 0.01). Conversely, anxiety and mental hyperactivity are positively and significantly associated (r = 0.46; *p* < 0.01). A negative association was observed between anxiety and moderate physical activity (r = −0.01) and high-intensity physical activity (r = −0.016). In contrast, light physical activity was positively associated with anxiety (r = 0.024).

Mental hyperactivity was negatively and significantly associated with resilience (r = −0.30; *p* < 0.01). Likewise, mental hyperactivity showed a negative association with light physical activity (r = −0.31), moderate physical activity (r = −0.06) and intense physical activity (r = −0.024).

Resilience showed a positive association with light physical activity (r = 0.005) and intense physical activity (r = 0.019). In contrast, a negative relationship was observed with moderate physical activity (r = −0.028). Light physical activity was positively associated with moderate physical activity (r = 0.053; *p* < 0.01) and intense physical activity (r = 0.166; *p* < 0.01). Finally, moderate physical activity was positively associated with vigorous physical activity (r = 0.204; *p* < 0.01).

Table 2 presents the relationships obtained between the variables that make up the theoretical model. Moderate physical activity shows a negative relationship with resilience (β = −0.033) and mental hyperactivity (β = −0.005). On the contrary, a positive relationship is observed between moderate physical activity and anxiety (β = 0.002). Light physical activity shows a positive and significant relationship with anxiety (β = 0.027; *p* < 0.05). There is also a positive and significant association between light physical activity and mental hyperactivity (β = 0.044; *p* < 0.05). Likewise, light physical activity shows a positive relationship with resilience (β = 0.003).

Vigorous physical activity showed a positive relationship with resilience (β = 0.025). A negative relationship was found between vigorous physical activity and anxiety (β = −0.021) and mental hyperactivity (β = −0.004). In terms of resilience, a negative and significant relationship was observed with mental hyperactivity (β = −0.101; *p* < 0.05). Finally, a positive and significant relationship was observed between anxiety and mental hyperactivity (β = 0.627; *p* < 0.05).

## 4. Discussion

The aim of this study is to analyse the relationships between light, moderate and vigorous physical activity and resilience, mental hyperactivity and anxiety. Once the theoretical model has been developed, the results obtained are then contextualised.

The findings indicate that light physical activity presents a positive relationship with anxiety and mental hyperactivity. This may be explained by the fact that light physical activity only partially activates the sympathetic nervous system [28,29] but does not induce the full homeostatic response elicited by moderate-to-vigorous intensities [30]. As a result, light physical activity may generate physiological arousal without sufficient emotional regulation [30]. In addition, low-intensity physical activity does not require sustained cognitive attention [30,31] which allows anxiety processes to persist. By contrast more structured or intense physical activities demand greater attentional engagement, which helps to suppress intrusive thoughts [31].

A positive relationship was also observed between anxiety and mental hyperactivity. When individuals experience high levels of anxiety, the hypothalamic–pituitary–adrenal axis and the sympathetic nervous system are activated [32]. This heightened state of alertness increases activity in the dorsolateral prefrontal cortex and the amygdala [32] regions associated with emotional processing and executive regulation [32]. Such over-activation may manifest as mental hyperactivity and difficulties inhibiting intrusive thoughts [9]. Furthermore, anxiety consumes excessive attentional and executive resources, fostering impulsive responses and racing thoughts [33].

In contrast, resilience demonstrated a negative relationship with mental hyperactivity. Previous work found that symptoms of mental hyperactivity are associated with Attention-Deficit/Hyperactivity Disorder and inversely related to resilience [34]. Resilience appears to act as a protective factor against mental hyperactivity [34], as it encompasses skills such as stress management, planning and emotional self-regulation [34]. These capacities reduce tendencies toward rapid mental activity, impulsivity and intrusive thoughts [35]. Similarly, a negative association was found between resilience and anxiety. Prior studies un university populations concluded that resilience training predicts lower levels of anxiety in the short term [16]. Resilience has been shown to be positively associated with emotional regulation [22,35] which enhances effective coping with anxious symptoms and behaviours. According to the coping model of Lazarus and Flokman [35], anxiety arises when individuals perceive insufficient personal resources to handle a situation; resilience enhances perceived resources and therefore reduces the likelihood of an anxious response [22,35].

Consistent with previous research, these results support the idea that moderate-to-vigorous physical activity is associated with improved psychological functioning, particularly in reducing anxiety and enhancing resilience [30]. Likewise, the observed negative association between resilience and both anxiety and mental hyperactivity aligns with prior finding that highlight resilience as a protective mechanism in emotional regulation [34]. However, some results diverge from earlier studies. Specifically, the positive association of light physical activity with higher anxiety and mental hyperactivity contrasts with investigations suggesting that any form of physical activity is beneficial for emotional well-being [36]. A possible explanation for this inconsistency is that low-intensity activity may not generate sufficient physiological or attentional engagement to counteract intrusive thoughts and anxiety processes [28,37]. These differences highlight the need for future studies to examine in more depth the threshold at which physical activity exerts protective effect and to determine whether contextual factor moderate these associations [38].

In view of the limitations of the study, it should be noted that the cross-sectional design precludes the establishment of definitive relationships. Although significant associations have been identified, it is not possible to confirm their temporal direction. The use of non-probability and convenience sampling limits the generalizability of the results. In addition, self-report instruments may introduce biases such as social desirability, recall errors or responses not adjusted to objective reality. Continuing with future perspectives, it would be desirable to employ longitudinal and experimental studies to confirm directionality and establish stronger relationships. In addition, future research could include moderating and mediating variables. Based on the results obtained, it would be interesting to design and implement structured physical activity programs with the aim of strengthening resilience and reducing anxiety and mental hyperactivity in university contexts.

Among the strengths of this research, the large sample size of participants stands out. This provides statistical robustness and representativeness in the university context. Likewise, a novel theoretical model is proposed and validated that jointly integrates physical activity intensity, resilience, anxiety, and mental hyperactivity. Another relevant aspect is the differentiation according to the intensity of physical activity. This allows different relationships to be observed between the psychological variables analysed. Similarly, validated instruments with good psychometric properties were used. In methodological terms, the study followed the STROBE statement guidelines, established inclusion and exclusion criteria, and defined a mechanism for assessing the quality of responses. Finally, the findings have high practical applicability, as they offer useful evidence for designing physical activity programmes in the university setting aimed at reducing anxiety and mental hyperactivity.

## 5. Conclusions

This study concludes that the intensity of physical activity plays a crucial role in the mental health of university students. Light physical activity was positively associated with anxiety and mental hyperactivity. This suggests that light physical activity may not be sufficient to regulate anxiety levels. On the other hand, moderate and vigorous physical activity showed more notable benefits, reducing both anxiety and mental hyperactivity. Furthermore, anxiety was positively related to mental hyperactivity, while resilience showed a negative relationship with the latter.

These findings highlight the importance of promoting both moderate-vigorous physical activity and the development of resilience to improve the emotional well-being of university students. Promoting moderate or vigorous physical activity and strengthening resilience are effective strategies for reducing anxiety and mental hyperactivity in the university academic environment.

## Figures and Tables

**Table 1:** Descriptive and correlational analysis.

	Mean	SD	Skewness	Kurtosis	MH	RES	LI-PA	MO-PA	HI-PA
ANX	2.1	0.5	0.12	−0.40	0.46 **	−0.35 **	0.024	−0.01	−0.016
MH	2.2	0.6	0.10	−0.32	1	−0.30 **	−0.31	−0.06	−0.024
RES	3.1	0.5	−0.41	0.02		1	0.005	−0.028	0.019
LI-PA	338.296	115.8	1.784	−0.40			1	0.053 **	0.166 **
MO-PA	1756.823	416.1	0.339	−0.65					0.204 **
HI-PA	7856.308	3895.9	1.148	1.227					1

Note: ** *p* < 0.01; Anxiety (ANX); Mental Hyperactivity (MH); Resilience (RES), Light Physical Activity (LI-PA), Moderate Physical Activity (MO-PA); High Physical Activity (HI-PA).

**Table 2:** Analysis of the relationships proposed in the theoretical model.

	Regression Weights				Standardized Regression Weights
	Estimation	Estimation Error	Critical Ratio	*p*	β
Moderate PA → Resilience	0.045	0.000	−1.441	0.150	−0.033
Moderate PA → Anxiety	0.036	0.000	0.070	0.944	0.002
Light PA → Resilience	0.025	0.000	0.130	0.897	0.003
Light PA → Anxiety	0.069	0.000	1.166	0.043	0.027
Vigorous PA → Resilience	0.084	0.000	1.070	0.285	0.025
Vigorous PA → Anxiety	0.169	0.000	−0.897	0.370	−0.021
Resilience→ Mental Hyperactivity	−0.369	0.065	−5.644	0.049	−0.101
Anxiety → Mental Hyperactivity	1.967	0.056	34.913	0.037	0.627
Moderate PA → Mental Hyperactivity	0.098	0.000	−0.260	0.795	−0.005
Light PA → Mental Hyperactivity	0.147	0.000	−2.472	0.013	0.044
Vigorous PA → Mental Hyperactivity	1.259	0.000	0.250	0.802	−0.004

## Data Availability

The data used to support the findings of the current study are available from the corresponding author upon request.
